# The biobehavioral family model with a seminarian population: A systems perspective of clinical care

**DOI:** 10.3389/fpsyg.2022.859798

**Published:** 2022-09-23

**Authors:** Kaitlin Smith, David Wang, Andrea Canada, John M. Poston, Rick Bee, Lara Hurlbert

**Affiliations:** ^1^Department of Counseling and Clinical Psychology, Medaille University, Buffalo, NY, United States; ^2^Fuller Theological Seminary, Pasadena, CA, United States; ^3^Rosemead School of Psychology, Biola University, La Mirada, CA, United States

**Keywords:** biobehavioral family model, seminarians, health, anxiety, depression, relationships, family, seminary students

## Abstract

Seminary students remain unstudied in the research literature despite their eminent role in caring for the wellbeing of congregants. This study aimed to conduct baseline analysis of their family of origin health, psychological health, and physiological heath by utilizing the Biobehavioral Family Model (BBFM) as a conceptual framework for understanding the associations between these constructs. Statistical analysis utilizing structural equation modeling provided support that the BBFM was a sound model for assessing the relationships between these constructs within a seminary sample. Additionally, seminarians were found to have higher rates of anxiety and depression when compared to the general population. Together, findings indicate that clinical care for seminarians may be best if implemented from a global systemic perspective.

## Introduction

To date, little research has been conducted on seminarian health. This population represents a sizable contingent of current and future clergy and religious leaders. Though not all religious leaders attend seminary, a seminary degree is still considered a valued milestone for the profession. It is difficult to get an accurate count of clergy serving congregations within the United States. Currently, there are an estimated 458,000 clergy members within the United States (Clergy Data USA, 2022) though data is not specific as to what roles they serve in (e.g., congregational ministry, chaplaincy) or the religion they subscribe to. Congregants as well as the general population often look to clergy and religious leaders as role models. Serving as role models for the community can place additional societal pressure on the clergyperson. Given these pressures, it is important to understand the status of health for seminarians prior to entering the ministry. Due to the limited research in this domain, to our knowledge, there are no specific treatment recommendations for seminarians. By investigating several aspects of health in conjuncture with each other, namely family health, psychological health, and physical health, we aim to develop a baseline assessment of seminarian health and lay the conceptual groundwork for developing therapeutic treatment recommendations.

### The biobehavioral family model

The Biobehavioral Family Model (BBFM) is a systems model that defines the interactions between one’s psychological functioning, social processes, and physical health (Wood, 1993). The BBFM was originally derived from the psychosomatic family model, which sought to account for the potential relationships between family health and emotional health, including psychosomatic illness (Minuchin et al., 1975). However, in recent years the BBFM has been adapted to include more facets of physical health, along with a more robust understanding of emotional health (Wood, 1993). Currently, the emotional health construct is defined as biobehavioral reactivity, which is commonly conceptualized as anxiety and depression. Accordingly, the model describes how family emotional climate is directly related to biobehavioral reactivity, which is directly related to disease activity. Thus, family emotional climate is indirectly related to disease activity as it is mediated through biobehavioral reactivity.

One of the original studies validating this revised version of the BBFM was Wood et al. (2006). This study examined the relationships between family emotional climate, as measured by family expressivity, childhood depression and asthma disease severity in children (mean age = 11.5, *SD* = 2.7). They found that all pathways in the BBFM were statistically significant, suggesting that the BBFM was a good fit for the data; negative family emotional climate was positively correlated to childhood depression, which was positively correlated with increased disease severity. Wood et al. (2008) found similar results when they included the construct of childhood attachment within the family emotional climate construct. Subsequent studies garnered additional empirical support for the model, with several studies incorporating romantic relationship health into the family emotional climate construct (Woods and Denton, 2014; Priest et al., 2015).

By utilizing the BBFM to investigate multidimensional health of seminarians, mental health professionals as well as leaders in theological education will be able to better understand the interrelatedness between these facets of health for these students. This may inform interventions that support the health of seminarians from a holistic perspective, potentially bearing positive impact on the future religious communities in which they serve. Though this model has not been studied within the seminarian population, research has supported the sufficiency of the BBFM for explaining relationships between these constructs in several populations including children and adults (Wood, 1993; Woods and Denton, 2014; Priest et al., 2015).

Given the current gap in the literature regarding the health and well-being of seminary students specifically, we are utilizing graduate students and clergy as related populations to provide a theoretical basis for our study. It is hypothesized that the transitional stress of graduate school and the stress of serving as role models in the community are part of what contributes to unhealth in these populations, respectively. Given that seminary students share these attributes with each respective population, we hypothesize their psychological and physical health will be similar.

### Family emotional climate

Although there is little empirical data on clergy members’ family of origin emotional climate, research has indicated that there are vulnerabilities for this population. Rickner and Tan (1994), for example, compared the family health of clergy with Christian high school teachers and public-school teachers. The researchers found that clergy perceived their families of origin as significantly less healthy overall compared to Christian high school teachers and public-school teachers (Rickner and Tan, 1994).

### Biobehavioral reactivity

Research has also pointed to clergy having higher rates of anxiety and depression compared to the general population. For example, Proeschold-Bell et al. (2013) surveyed Methodist clergy in North Carolina. They found that the depression rate among those surveyed was 8.7%, which was significantly higher than the 5.5% rate of the national survey data. Similarly, Knox et al. (2002) surveyed a random sample of Roman Catholic Priests in the United States. Of the randomized sample, 20% met the cutoff for depression based on the Center for Epidemiological Studies-Depression (CES-D) scale by Radloff (1977). Also 15% of Catholic clergy met criteria for significant state anxiety and 20% met criteria for significant trait anxiety according to the using the State–Trait Anxiety Inventory (STAI) Form Y (Spielberger and Krasner, 1988). Though the CES-D and STAI do not necessarily constitute a formal diagnosis of Generalized Anxiety Disorder or Major Depressive Disorder, according to the Diagnostic and Statistical Manual (DSM) they are both accurate in pinpointing clinically significant levels of anxiety and depression (American Psychiatric Association, 2013).

Research has also investigated the attitudes that clergy hold towards mental illness. Payne and Hays (2016) performed a qualitative analysis of dialogues among interdenominational clergy on the topic of mental health within the context of a clergy social networking group. They found that the clergy who responded to a post on mental health endorsed a spectrum of views regarding mental health. On one end of the spectrum, some clergy believed that mental health concerns were a spiritual matter and likely a result of demonic oppression, diminishing values/worldliness, and lack of trusting in God. On the other end of the spectrum were clergy who held a medical model understanding of mental health. Overall, most clergy seemed to hold views that were somewhere in the middle (e.g., psychology could be helpful but healing should ideally include God). Given these varying views on mental health, it is possible that there are clergy who are struggling with mental health difficulties but are afraid that admitting so would be in tension with their religious beliefs or beliefs espoused by fellow clergy members.

Clergy’s perception of mental illness can vary based on race and religion affiliation (Payne, 2009). Payne (2009) surveyed a heterogeneous sample of Protestant pastors regarding their beliefs on the etiology of depression. Payne found that white pastors were more likely to believe that “depression was a biological mood disorder” compared to African American pastors. Additionally, African American pastors were more likely to believe that “depression is hopelessness that happens when one does not trust God” when compared to white clergy. In regards to religious affiliation, Payne found that Pentecostal and non-denominational pastors were more likely to endorse spiritual causes of depression (e.g., depression is due to a lack of faith in God.

With regards to the mental health of graduate students, Lipson et al. (2016) investigated the prevalence of depression, anxiety, suicidality, and self-injury among graduate students. They found that 13% of all graduate students screened positive for an anxiety or a depressive disorder. Additionally, results indicated that for both masters and doctoral level students, mental health problems were significantly higher among students studying a discipline within the humanities. Although religion/pastoral studies were not specifically studied, it could be argued that these disciplines bear meaningful similarity to the humanities. Barton (2012) found similarly high rates of anxiety and depression in graduate students. Of a sample of 4,477 graduate students, 14% screened positive for depression (i.e., major depressive disorder or other depressive disorder), 9.5% screened positive for anxiety disorder (i.e., generalized anxiety disorder or panic disorder), and 4.4% screened positive for both. Taken together, these findings raise the possibility of mental health difficulties among graduate student seminarians.

### Disease activity

Research has also indicated that clergy tend to have poor physical health. Lindholm et al. (2016), for example, studied the physical health of clergy in Kansas compared to the general population of Kansas. Out of the 150 clergy who participated, 77.4% reported weight and height values that classified them as obese or overweight. Additionally, clergy self-reported higher rates of chronic diseases than the general Kansas population. Interestingly, when asked to describe their health, 93.7% of clergy described their health as either good, very good, or excellent. It appears that, despite being in objectively poor physical health, clergy members subjectively believed they were in good health. Other studies have found similar results indicating poor physiological functioning of clergy including higher rates of obesity, diabetes, arthritis, high blood pressure, angina, and asthma compared to the general population (Proeschold-Bell and LeGrand, 2010, 2012).

Graduate students also appear to have similarly poor physical health. Rummell (2015) conducted an exploratory study regarding the health of 119 psychology graduate students. Students reported experiencing headaches (78.8%) back pain (61%) irritable bowels (57.5%), upset stomach (48.6%), and weight gain or loss (38.4%). This study also collected data on the prevalence of mental health symptoms and found a significant positive correlation between the number of physical symptoms reported and the number of mental health symptoms reported. Correspondingly, Melnyk et al. (2016) found that nearly 40% of the graduate students they surveyed were overweight or obese, 19% had elevated cholesterol levels, and only 44% were getting the recommended 30 min of exercise 5 days per week. Additionally, 28% of students had elevated anxiety and 41% of students reported elevated depressive symptoms. Finally, increased mental health symptoms were inversely correlated with healthy lifestyles.

## The present study

### Purpose

The purpose of this study is to establish a baseline of seminarian health in the literature and to test the sufficiency of the BBFM in explaining the relationships between family of origin emotional climate, psychological health and disease activity. This population remains understudied; yet is on the precipice of being responsible for the spiritual care of their congregants. Given that congregants tend to look to their pastors as role models, it is important to understand the health of this population. By understanding seminarian health from a systemic point of view, we hope to provide preliminary treatment recommendations for this population.

**Figure 1:**
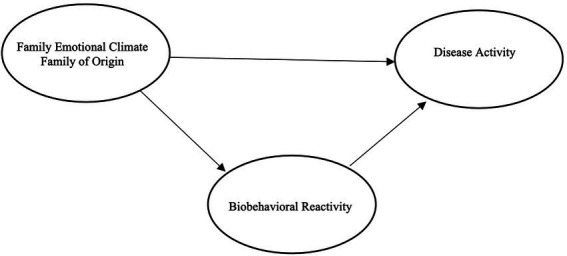
Hypothesized model with direct path.

**Figure 2:**
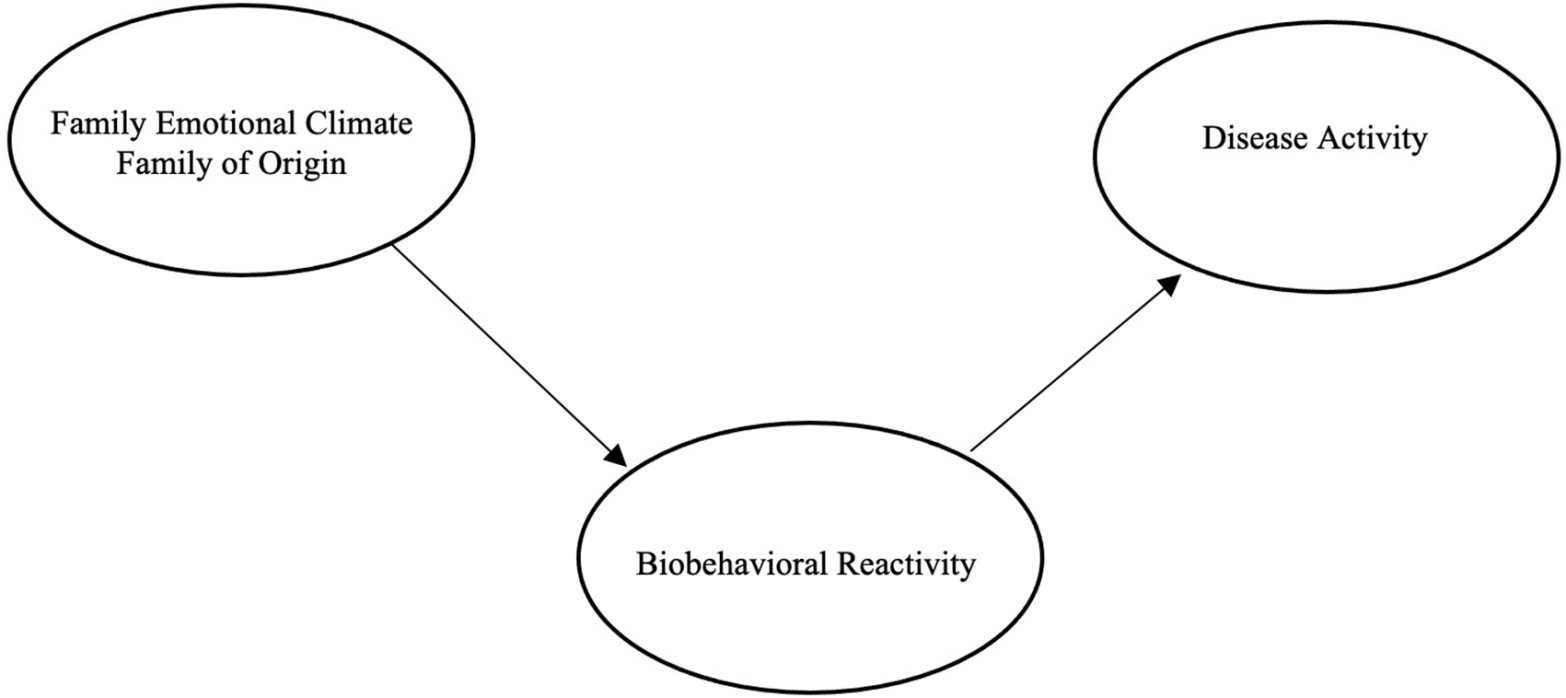
Hypothesized model with meditation.

Reflective of the body of literature, we hypothesize that seminarian students will have poorer family of origin emotional climate, higher levels of anxiety and depression, and higher levels of disease activity as evidenced by poorer physiological health. Given the previous literature supporting the BBFM, we further hypothesize that this will be a sufficient model in explaining the relationships between these constructs (Wood et al., 2008; Proeschold-Bell et al., 2013; Woods and Denton, 2014; Priest et al., 2015). Namely, we hypothesize that among seminary students, poorer family of origin emotional climate will be related to poorer psychological health, which will be related to poorer physiological health (see Figure 1). In concurrence with previous research, we will test a direct relationship between family of origin emotional climate and disease activity; however, we hypothesize that that the relationships will best be accounted for by a mediation though biobehavioral reactivity (see Figure 2).

This study is a part of a larger study made possible by a grant from the Lilly Foundation to explore the multifaceted impact of seminarian debt on the wellbeing and functioning of seminary students. This study was approved by the Protection of Human Rights in Research Committee (PHRRC) at Biola University.

## Methods

### Participants

Participants were recruited from a large, theologically conservative, interdenominational Evangelical seminary in Southern California *via* email. The sample included both current students and recent alumni (i.e., no later than 5 years post-graduation). The total number of participants in the sample was 120 and the average age of participants was 29.5 (*SD* = 7.04, range 21–54). Of the total participants, 30.8% were white (*n* = 37), 1.7% were Black (*n* = 2), 6.7% were Hispanic or Latino (*n* = 8), 59.2% were Asian or Pacific Islander (*n* = 71), and 1.7% described themselves as “other” ethnicity (*n* = 2). Additionally, 69.2% (*n* = 83) were male and 37% (*n* = 37) were female. Of the total participants, 58.3% (*n* = 70) were enrolled in the M.Div. program, 39.2% (*n* = 47) were enrolled in an M.A. program, and 2.5% (*n* = 3) were enrolled in a Masters of Theology degree. Finally, 51.7% (*n* = 62) of the students were single, 30.8% (*n* = 37) were married, 4.2% (*n* = 5) were engaged, 9.2% (*n* = 11) were dating exclusively, 0.8% (*n* = 1) were dating non-exclusively, and 3.3% (*n* = 4) reported their relationship status as “other.”

### Measures

In addition to the measures outlined below, the participants were first asked to report basic demographic information. This information included variables such as sex, age, race, religion and relationship status.

#### Family emotional climate

The seminarian’s family of origin emotional climate was measured by the Satisfaction with Family Life (SWFL) Scale and the Parental Caregiving Style Questionnaire, both the mother and father versions. We chose these measures to gather global data that assessed both current attitudes towards family of origin as well as more historical attitudes (e.g., attachment style).

The SWFL scale assesses respondents’ current feelings about their family of origin. It is composed of five Likert-type items, which require the respondent to choose from 1 = strongly disagree to 7 = strongly agree with global statements about family life, for example “I am satisfied with my family life” (Zabriskie and Ward, 2013). Scores range from 5 to 35 where higher scores indicate more satisfaction with family life. Zabriskie and Ward (2013) validated the SWFL scale on both parents and adolescents, among diverse family types, including families with a child with a disability, biracial adoptive families, single parent families, as well as families of various cultural backgrounds. Internal consistency of this measure was strong with Cronbach’s alpha ranging from *α* = 0.91 to *α* = 0.94. Test–retest reliability was established within a sample of undergraduate students (*r* = 0.87, *p* < 0.001). Validity was established though comparison of SWFL scores between general samples of families and families that have a known stressor, such as a child in mental health treatment. The SWFL was able to detect significant differences (*t* = 8.5, *p* < 0.01) between these two populations. Therefore, the SWFL scale is considered to be psychometrically sound.

The Parental Caregiving Style Questionnaire (Hazan and Shaver, 1986) was used to assess the seminarian’s perceived childhood attachment to both mother and father. With this measure, participants are presented with three short paragraphs describing avoidant, secure, and anxious attachment styles, respectively, (cf. Ainsworth et al., 1978). In keeping with previous research (e.g., Granqvist and Hagekull, 1999; Granqvist and Kirkpatrick, 2004), participants were asked to rate on a Likert scale (1 = *Strongly Disagree* to 6 = *Strongly Agree*) how much each paragraph described their childhood relationship with their mother. Participants were asked to rate the paragraphs a second time regarding perceived childhood relationship with father. In the current study, only secure attachment scores were utilized for data analysis (cf. Granqvist and Kirkpatrick, 2004). This measure has been used in previous research exploring the impact of perceived childhood attachment on later spiritual/religious functioning, and scores have been significantly related to variables such as religiosity and religious change/conversion (Granqvist and Hagekull, 1999).

Two additional questions were developed specifically for this study with the intent to assess the degree of conflict over seminarian career choice. Specifically, participants were asked to use a 9-point Likert scale (1 = *Not severe at all*; 9 = *Very severe*) to rate severity of conflict with family on the following two items: “Disagreement between family members (not including your spouse/romantic partner) and yourself concerning your career choice?” and “Disagreement between family members (not including your spouse/romantic partner) and yourself concerning desirable characteristics of a career?”

#### Biobehavioral reactivity

Biobehavioral reactivity was measured by the CES-D-10 and the Generalized Anxiety Disorder-7 (GAD-7). For these respective scales, higher scores indicate more significant symptoms of anxiety and depression. The CES-D-10 has well researched psychometric properties (Andresen et al., 1994). Within this study, test–retest reliability after 12 months was significant (*r* = 0.59, *p* < 0.01, *N* = 1,006). Validity was established by correlation with measures of self-assessed stress (*r* = 0.43). Finally, convergent validity was established by negative correlation with positive affect scores (*r* = −0.63). Taken together, the CES-D-10 is considered a valid assessment of depression.

The GAD-7 has been demonstrated to have sufficient convergent validity by its correlation with the Beck Anxiety Inventory (*r* = 0.72) (Spitzer et al., 2006). Additionally, the GAD-7 has good internal consistency (Cronbach’s *α* = 0.92) and good test–retest reliability (intraclass correlation = 0.83). This measure requires participants to rate the frequency at which they experience Generalized Anxiety Disorder symptoms over the last 2-weeks.

#### Disease activity

Disease activity was measured by the Health Promoting Lifestyle Profile (HPLP II), the self-reported health measure, and sleep questionnaire. For each of these measures, higher scores indicate a less healthy lifestyle. The HPLP II is originally a 6-factor measure that assess for dimensions of healthy lifestyle choices (Walker et al., 1987). Each of these six factors were shown to have good internal consistency ranging from Cronbach’s *α* 0.702–0.904 with an overall internal consistency of Cronbach’s *α* = 0.922. The HPLP II requires participants to select how frequently they engage in healthy lifestyle choice behaviors. For this particular study only the Diet, Exercise, and Stress Management factors were used for brevity’s sake. Given that only the healthy behavior items will be used it will be referred to as the *sum of health behaviors* from this point forward.

The self-reported health measure was also utilized to assess for disease activity (Sargent-Cox et al., 2008). This measure assesses self-reported health on the global level, age-comparatively, and self-comparatively, each measured by one item. The measure can yield a total score for statistical analyses, or the scores and distribution on each item can be analyzed separately. Criterion and sensitivity/specificity for this measure was established by distinguishing self-reported health between various age groups, where older adults had scores indicative of poorer health, and younger participants were rated as healthier. This measure is utilized in statistical analysis, in addition to ordinal ranking understanding of self-reported health. Therefore, it is helpful to also analyze the response patterns of the participants.

For the sake of brevity, two items were taken from the Pittsburg Sleep Quality Index (PSQI; Buysse et al., 1989) to assess how many hours of sleep participants get each night. The PSQI is a 19-item self-report measure. The measure as a whole has established good psychometric properties, however there are no studies regarding the individual items.

### Procedure

Participants were contacted *via* email for recruitment by sending a direct link to the online survey to complete all self-report measures. Upon completion of the survey, data was deidentified by providing each participant a generic unique identifier. Data analysis was conducted using Statistical Package for the Social Sciences (SPSS) version 25 and Stata version 12. First, descriptive statistics and zero-order correlations were generated using SPSS. The descriptive statistics for the SWFL scale, GAD-7, CES-D, and self-reported health were used to establish baseline statistics for the family emotional climate, psychological health and physical health of seminary students. Finally, structural equation modeling analysis was conducted using Stata. Several goodness of fit indices was subsequently examined to test the adequacy of the proposed models. Specifically, in keeping with previously published guidelines established by Kline (2011), a model was considered a good fit for the data if Chi-Square (*χ*^2^) was *p* > 0.05, root mean squared error of approximation (RMSEA) was <0.05 90% CI left 0.00, upper <0.10, and comparative fit index (CFI) was >0.95.

## Results

### Data preparation

A missing variable analysis was completed on the available data. Out of the original 126 participants, six were missing 75% or more of the data. Accordingly, these six participants were removed from the analysis. The remaining 120 participants were not missing any data. All variables were screened for skewness and kurtosis to ensure normal distribution. Ideal cut off scores for both were ± 3. All variables fell within the desired range. For the SEM analysis, utilized sample size guidelines outlined by Schreiber et al. (2006). They suggest a 10:1 participant to estimated parameter ratio. We proceeded with our SEM analysis after it was determined that our ratio was 10.91–1.

### Family emotional climate

The SWFL scale was utilized to measure family of origin satisfaction. In the original publication of the measure, the normative sample consisted of families from the general population (*N* = 898) who yielded a mean of 24.47 where higher scores indicated more satisfaction with family life (Zabriskie and Ward, 2013). Our sample yielded a mean of 23.01 (*SD* = 8.02). In order to compare our sample with the original normative study, a one-sample t-test was utilized. The results indicated that our sample trended toward being less satisfied than the general public *t*(119) = −1.98, *p* = 0.050, potentially warranting future attention to seminarian family life in the research literature.

### Anxiety and depression rates

The widely used interpretive thresholds for scores on the GAD-7 are scores of 5, 10, and 15 to indicate mild, moderate, and severe anxiety, respectively, (Spitzer et al., 2006). The mean GAD-7 score for our sample was $\bar{x}$= 5.85, *SD* = 4.49. Our results indicate that the average seminarian in this study was experiencing mild levels of anxiety. In looking at the distribution of the GAD-7, 49.2% of the participants had a GAD score ≥ 5 (mild anxiety), 19.7% had a GAD score ≥ 10 (moderate anxiety), and 4.2% had a score ≥ 15 (severe anxiety).

Depending on the demographic qualities of the sample (i.e., age and health) a cutoff score of ≥8 or ≥ 10 on the CES-D-10 is recommended to indicate clinically significant depressive symptoms. The mean for our sample fell above both recommended cutoff points with $\bar{x}$= 10.6, *SD* = 5.75. This indicated that the seminarian sample in this study was experiencing clinically significant levels of depression, especially when compared to the general population. Of our seminary sample, 55.8% had CES-D-10 scores ≥8 and 45% had scores that were ≥ 10. This indicated a high frequency of depression within this seminarian sample.

### Disease activity

**Figure 3:**
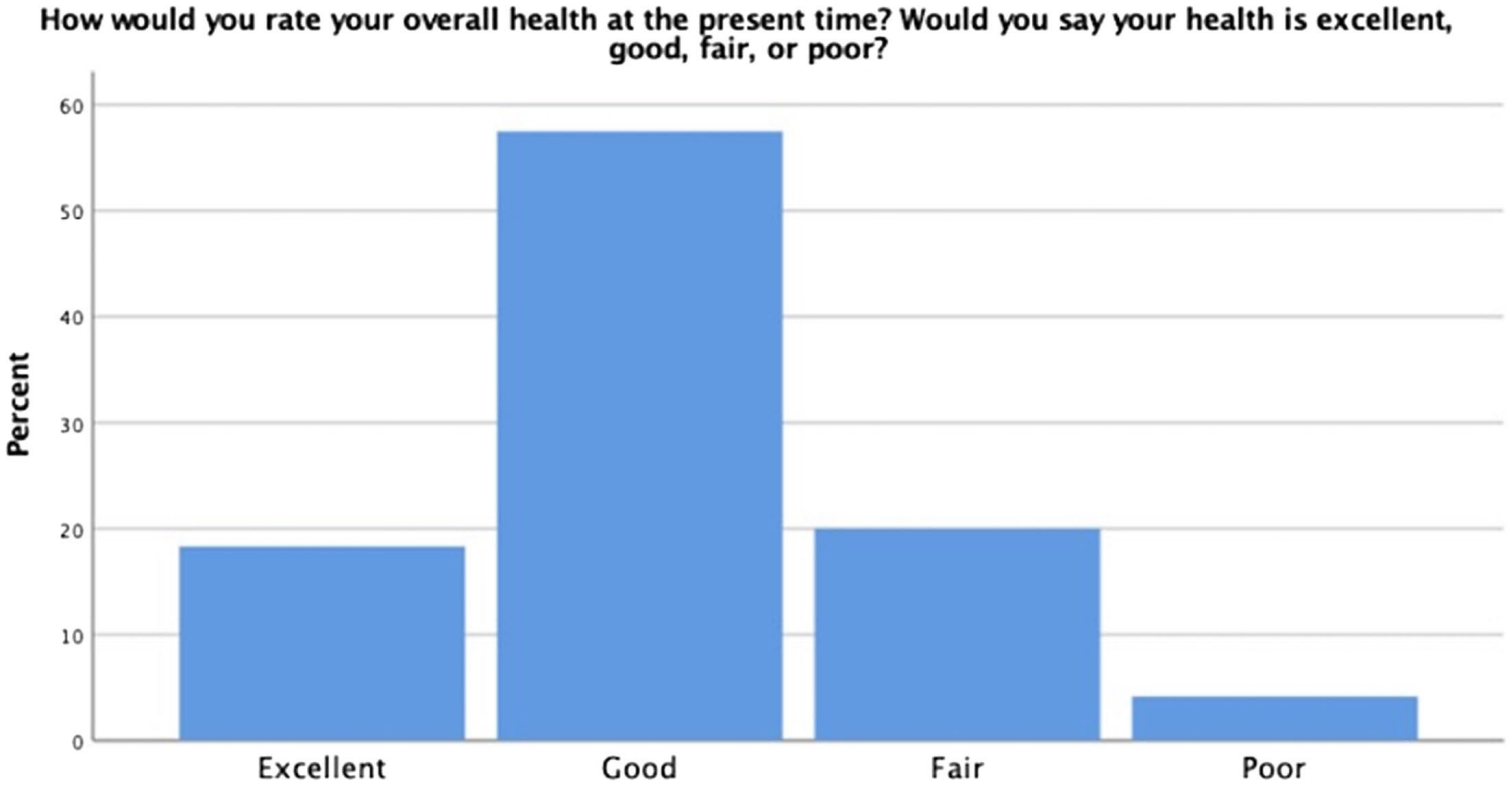
Global self-reported health.

**Figure 4:**
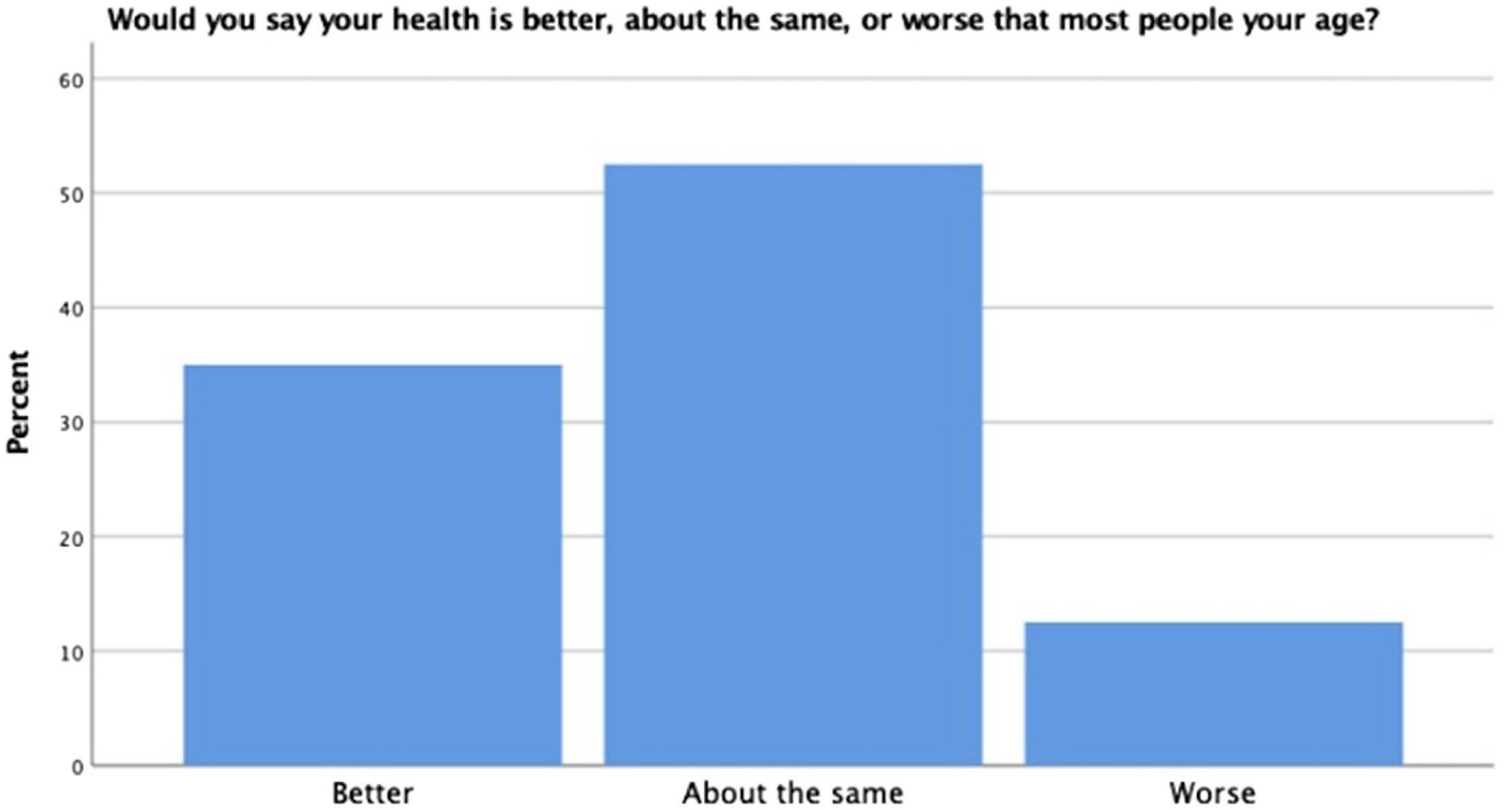
Age comparative health.

**Figure 5:**
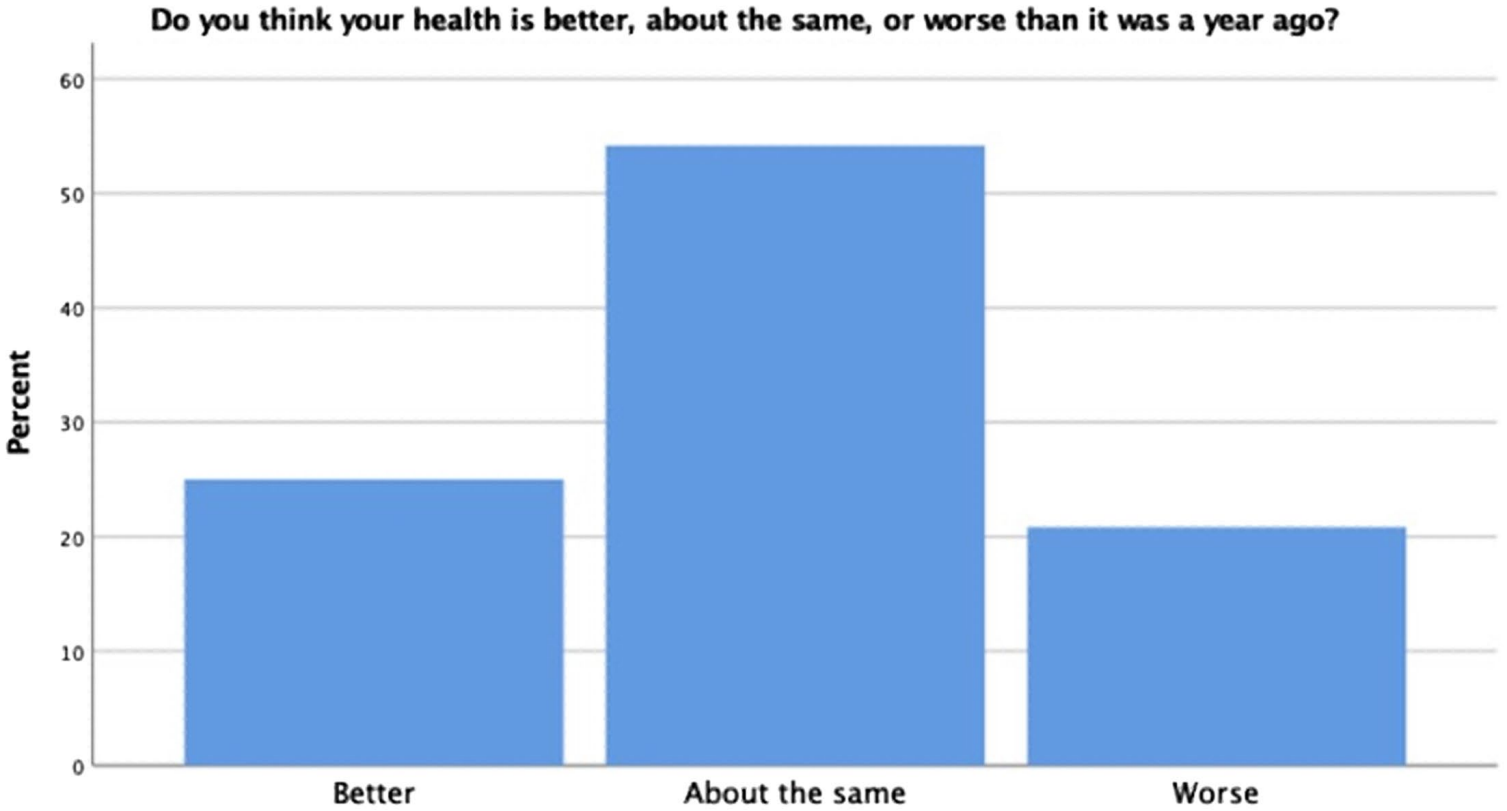
Self-comparative health.

On the self-reported health measure, a robust portion of our sample reported themselves to be of relatively good health (57.5%); however, others reported their health as excellent (18.3%), fair (20%), and poor (4.2%) (see Figure 3). When participants were asked to rate their health in relation to others their age, the majority indicated that they considered themselves about the same at others (52.5%). The remaining participants rated themselves as being in better health than others their age (35%) and worse than others their age (12.5%) (see Figure 4). Finally, when asked if they thought their health was better, the same, or worse than a year ago, most participants indicated their health was the same (54.2%) (see Figure 5). The remaining participants indicated their health was either better than a year ago (25%) or worse than a year ago (20.8%). Taken together, the results of self-reported health indicate that seminarians are in relatively good health.

### Zero-order correlations

Within the family emotional climate latent variable, disagreement with family over career choice was negatively correlated with satisfaction with family life (SWFL) at the *p* < 0.01 level. Disagreement with family over career choice was not significantly related to secure attachment with either the mother or father. SWFL was significantly related to all variables within the family emotional climate variables at the *p* < 0.01 level. Secure attachment to mothers as well as secure attachment to father were only significantly correlated with satisfaction with family life.

The biobehavioral reactivity latent variable was comprised of two observed variables, the GAD-7 and CES-D-10. These two observed variables were highly correlated (*r* = 0.75, *p* < 0.01). The disease activity latent variable consisted of the following observed variables, sum of health behaviors, Pittsburg sleep questionnaire and self-reported health. Within this construct all variables were correlated at the *p* < 0.01 level.

Of note, the two items taken from the PSQI were shown to correlate with all variables of biobehavioral reactivity and disease activity in the expected directions at the p < 0.01 level, providing some evidence of convergent validity. They were not significantly correlated with measures of family of origin emotional climate providing some evidence of discriminant validity. Taken together, these findings provided preliminary evidence of validity for this two-item assessment.

Outside of correlations within each latent variable construct, there were several other significant correlations of note. The CES-D-10 was significantly correlated with all remaining variables within the analysis in the expected directions. This indicated that higher levels of depression were significantly related to worse qualities of family life and worse overall health. The GAD-7 was significantly correlated with disagreement with family, satisfaction with family life, secure attachment to mother, health behaviors, and sleep quality in the expected directions. Satisfaction with family life was significantly correlated with health behaviors indicating that seminarians who were more satisfied with their family lived healthier lifestyles (see Table 1).

**Table 1:** Correlations between all variables.

Hypothesized latent variable		1	2	3	4	5	6	7	8	9
Family of origin Emotional climate	1. Disagree w/Fam	–								
	2. Satisfaction FL	−0.31^**^	–							
	3. Mom Secure Att	−0.12	0.52^**^	–						
	4. Dad Secure Att	−0.07	0.28^**^	0.16	–					
Biobehavioral reactivity	5. CESD	0.29^**^	−0.42^**^	−0.26^**^	−0.19^*^	–				
	6. GAD	0.24^**^	−0.42^**^	−0.26^**^	−0.11	0.75^**^	–			
Disease activity	7. Health Bx	0.08^**^	−0.21^*^	−0.15	−0.03	−23^*^	−20^*^	–		
	8. Sleep	0.15	−0.18	−0.12	0.04	0.36^**^	0.39^**^	0.30^**^	–	
	9. SR Health	0.17	−0.15	0.03	0.01	0.23^*^	0.16	0.33^**^	0.27^**^	–

Health bx, Sum of health Behaviors Measure; SR Health = Self-Reported Health.

* *p* < 0.05;

** *p* < 0.01.

### Structural equation model

**Figure 6:**
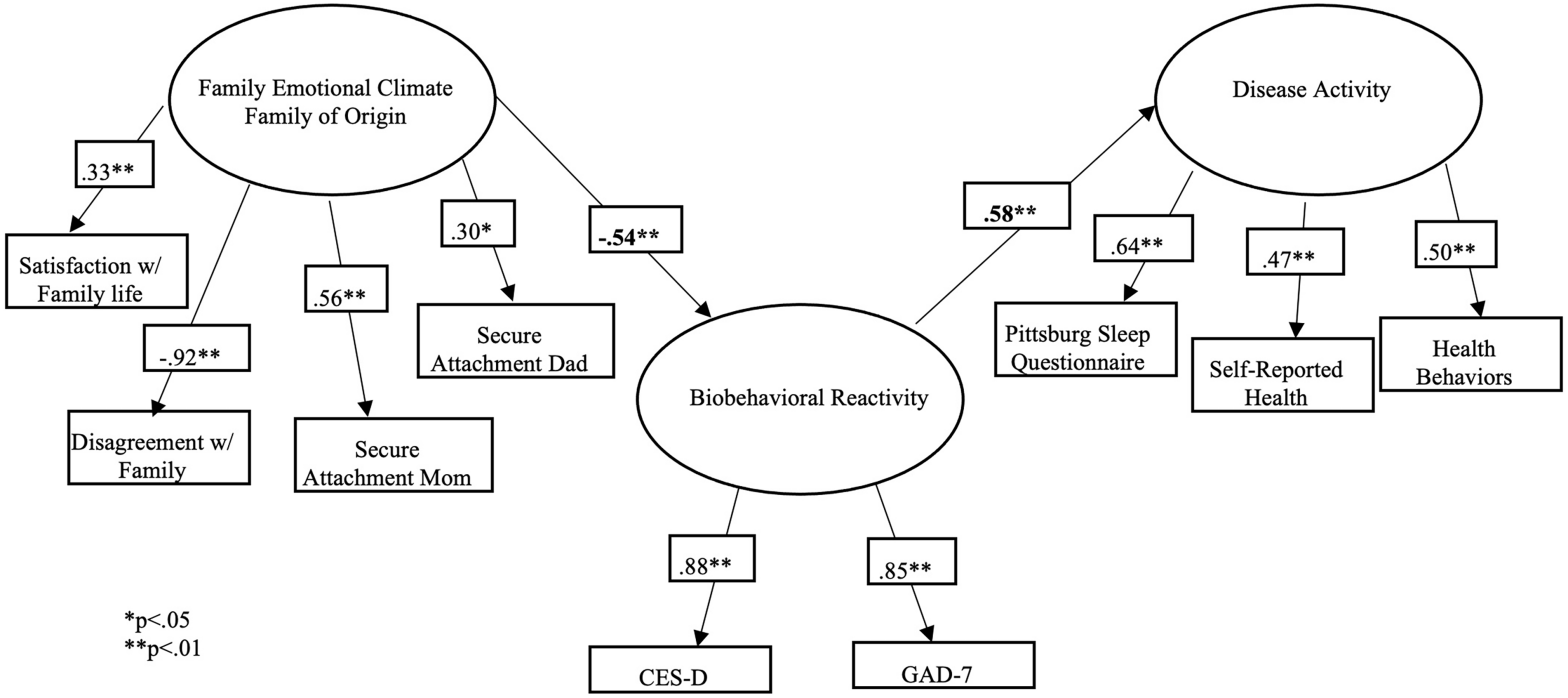
Structural equations model results.

SEM analysis was conducted to test the hypothesized model. The model was originally tested with a direct path between family emotional climate and disease activity and indirect path as mediated by biobehavioral reactivity. Notably, the direct path from family of origin emotional climate to disease activity was not statistically significant. The 95% confidence interval for the indirect effect based on 10,000 Monte Carlo replications did not include zero (−0.50 to −0.13), and the Sobel test (normal theory test) also indicated that the mediation effect was significant (Z = −3.20, *p* < 0.001). Accordingly, the results provided evidence that family of origin emotional climate was significantly and indirectly related to physiological health through psychological health. Therefore, the direct path was removed, resulting in a more parsimonious model. For this remaining model, overall, goodness of fit indices indicated good fit to the data (*χ*^2^ = 19.356, *p* = 0.78, RMSEA = 0.00, 90%CI (0.00, 0.50), CFI = 1.00) with all remaining paths statistically significant at the *p* < 0.01 level and all factor loading significant at the <0.05 level in the expected directions. Results indicate that the BBFM is a good fit for explaining the relationships between the variables of the model (see Figure 6).

## Discussion

### Family emotional climate

To establish a baseline for the family health of seminary students, the results from the SWFL scale were compared to previous research. Though the results were not statistically significant, (*t* (119) = −1.98, *p* = 0.05) our sample demonstrated a trend toward less satisfaction with their family life than the original general population normative sample. Given these close results, further research regarding family of origin health and satisfaction of seminary students is needed.

### Biobehavioral reactivity

One of the most significant findings was the rate of anxiety and depression within this population. Notably, this finding was congruent with our hypotheses. In our sample, we observed a mean GAD-7 score of 5.85. According to the normative data from the original publication, the average seminarian is experiencing mild levels of anxiety. It is also important to note that although the mean of the current sample falls within the mild range (49.2%), almost 20% of participants were experiencing moderate levels of anxiety and 4.2% were experiencing severe anxiety for which further evaluation is recommended.

Results also indicated that our seminarian population was experiencing high rates of depression $\bar{x}$= 10.6, *SD* = 5.75. Previous research indicates that scores ≥8 or ≥ 10 are considered clinically significant (Andresen et al., 1994). When determining which cutoff score to utilize, demographic information is typically referenced. For example, samples that are comprised primarily of participants who are older or more medically compromised should utilize the ≥10 cutoff, as their scores might have been over inflated by these factors. Given the young age of our sample ($\bar{x}$= 29.5, *SD* = 7.04) the ≥8 cutoff would likely be more appropriate. With that in mind, 55.8% of our sample had scores ≥8. This indicated that more than half of the seminary students within this study were experiencing clinically significant depression. Even if utilizing the more conservative cutoff score of ≥10, 45% of the sample were experiencing clinically significant depression. This was an alarming statistic for this population, particularly when keeping in mind that the original sample had a mean score of 4.7.

It is important to consider previous research on clergy attitudes when interpreting the results of this study. Payne and Hays (2016) found that clergy endorsed varying views on mental health. Some clergy indicated that they believed that mental health issues were simply matters of spiritual unhealth and therefore should be healed through spiritual methods such as prayers. Some clergy endorsed a medical model, which encompasses empirically based diagnoses and treatment. The majority of clergy endorsed views that were somewhere in the middle, in which they believed in the role of psychology in diagnosis and treatment, but also believed that treatment should include spiritual elements. Payne (2009) also found that the clergy’s perception of mental health varied based on race as well as religious affiliation. White clergy were more likely to endorse beliefs that depression was a biological mood disorder than African American clergy. Additionally, Pentecostal and non-denominational clergy were more likely to endorse spiritual causes of depression than mainline Protestants. Given that our sample was from a theologically conservative interdenominational seminary, it is likely they hold more conservative views towards mental health. Our observed findings of higher rates of anxiety and depression symptoms are not to be taken lightly as it is possible that they have been underreported. Seminaries should be aware of the mental health challenges that many of their students are facing. Ideally, psychoeducation and psychological resources should be made readily available to students. Given that the majority of clergy believed that mental health care should include God, seminaries are uniquely positioned to offer this type of integrative care for their students.

These findings regarding the prevalence of anxiety and depression have significant implications not just for seminarians but for clergy. Given that clergy also have higher rates of anxiety and depressions according to previous research, it is possible that these mental health disorders begin during or even prior to seminary and continue throughout the span of a clergy member’s career (Knox et al., 2002; Proeschold-Bell et al., 2013). This further emphasizes the need for psychological services during seminary to provide early intervention.

### Disease activity

Self-reported physical health was also investigated to establish a baseline for the literature on seminarian health. Results of each item were looked at individually as done in previous studies (Sargent-Cox et al., 2008). Most seminary students reported that they were in good health, they were of similarly good health as others their age, and are as healthy as they were a year ago. Though these are positive results for this population, they must be interpreted within the context of previous literature. Lindholm et al. (2016) found that despite clergy being in more objectively poor health than the general population, they rated themselves as relatively healthy. Given the remainder of the disease activity variables were select items taken from full scales; a more thorough analysis of physiological health could not be completed. Although our sample reported being healthy, unless compared to other more objective measures of physical health, the true physiological health of this population remains understudied.

An interesting finding from correlational analysis was the significant correlation between satisfaction with family life and health behaviors. This positive correlation indicated that individuals who were more satisfied with their family of origin life tended to live healthier lifestyles. It is unknown why overall family life satisfaction is significantly related to overall health behaviors; however, it is an area for future research.

### Structural equation model

The structural equation model testing the BBFM was considered an excellent fit for the data with all paths being significant at the *p* < 0.01 level. This provides additional support for the utility of this model across populations as it appears to function similarly when compared to previous studies (Wood, 1993; Woods and Denton, 2014; Priest et al., 2015). In our sample, healthier family of origin emotional climate was associated with less anxiety and depression, which was associated better physiological health. The relationship between family of origin emotional climate and disease activity is mediated though biobehavioral reactivity. This is congruent with our hypotheses that the BBFM would be a good fit for explaining the relationships between family emotional climate, biobehavioral reactivity, and disease activity. Given the cross-sectional design of the study, there is not sufficient evidence to indicate that there is a causal relationship between these variables. That said, given the relatedness of these constructs, preliminary treatment implications can be made.

These findings provide some empirical support for the provision of psychological services from a systemic conceptual framework. Concerns regarding family, mental health struggles and physiological illness can each be conceptualized and treated independently; however, this may not be the best practice for this population. Psychotherapy approaches such as CBT or psychopharmacological interventions can help reduce anxiety and depression, which may in turn reduce disease activity. However, this approach may not address the root of the problem. A comprehensive approach would likely be beneficial for these students. Clinicians working with this population may wish to address family emotional health for example, by focusing on attachment concerns. If treatment fails to include this aspect of the seminarian’s life, underlying problems may remain unaddressed and will likely reactivate symptoms of biobehavioral reactivity and disease activity.

### Limitations

The authors acknowledge that a significant limitation of this study is the lack of emphasis on culture and diversity. Previous research indicates that there are racial differences in physiological and mental health specifically within the clergy community (Case et al., 2018). For example, Case et al. (2018) found that even when controlling for socioeconomic status, Black clergy had significantly more hypertension than their White clergy counterparts. Additionally, White clergy had significantly more depressive symptoms compared to Black clergy. Research also indicates that race has a significant impact on how clergy view mental health (Payne, 2009). Though there is not research on the impact of race within seminary students, the available body of research within clergy does indicate race may have an impact on their mental and physical health. Future research would benefit from further exploring the impact that race has on the health of seminarian students and its impact on the BBFM.

An additional limitation of this study was that the sample was taken from a single seminary. Given that all participants were studying at the same seminary; it is likely that they hold similar religious views. Previous research, indicates that religious views have an impact on opinions of mental health (Payne, 2009; Payne and Hays, 2016). A more diverse seminary sample may help to further illuminate the role that biobehavioral reactivity plays within the BBFM. Additionally, our sample was limited geographically. All participants attended a seminary located in Southern California. Given that this area is relatively well known for being more health conscious, this also might have restricted the range of the disease activity variable. To help establish generalizability to the United States; a more geographically diverse sample is recommended. Finally, our study was cross-sectional in design and reliant on self-report measures. This limits the predictive nature of our findings and subjects them to performance bias.

### Directions for future research

Future research should further assess the quality of seminarian’s family of origin emotional climate. Our study indicated that SWFL was close to being significantly worse in this population compared to the normative sample, *t*(119) = −1.98, *p* = 0.050. Given these results, future research should look at the overall health and satisfaction of seminary student’s family of origin. The majority of seminary students reported they were of good health, however previous research indicates that participants are not always able to accurately report their own health (Lindholm et al., 2016). Therefore, future research should focus on comparing seminarian’s self-reported health to objective measures. Previous research has indicated that race and religious affiliation impact clergy’s views on mental health (Payne, 2009; Payne and Hays, 2016). As such, future research should explore the impact that these characteristics have on the BBFM constructs. Finally, a current trend in the literature is clergy burnout (Miner, 2007; Proeschold-Bell et al., 2015; Adams et al., 2017). Though biobehavioral reactivity as it is conceptualized in this study is not the same as burnout, there are multiple similarities between the two constructs. Research has even identified that burnout can lead to anxiety and depression (Miner, 2007). A worthy path of exploration would be to use the BBFM to look at the construct of burnout in clergy from a systemic perspective.

## Conclusion

The current study helped to establish a baseline of seminarian family of origin emotional climate, psychological and physiological health. Results indicated that seminarians were relatively satisfied with their families of origin. Unfortunately, seminary students were found to have clinically significant symptoms of anxiety and depression, which calls for intervention services. Finally, most seminarians reported being in good health; however, more research should be done to compare self-reported health to objective health.

The BBFM proved to be a good fit in explaining the relationships between family emotional climate, biobehavioral reactivity, and disease activity. Given these results, seminaries clinical providers, and spiritual leaders working with this population may benefit from taking a systemic approach to clinical care. This includes assessing and treating family health in addition to psychological and physiological health. Given previously studied attitudes that clergy hold towards mental health, interventions for this population should also include an aspect of spiritual healing (Payne, 2009; Payne and Hays, 2016). Seminaries and religious governing bodies are uniquely situated to provide integrative approaches to mental and physical health care. Therefore, these institutions would benefit from closely with mental health providers to determine integrative approaches to systemic treatment. Clinicians working with seminarians should also be aware of the unique stressors that await this population as they enter ministry. As indicated by this study, a systemic approach incorporating all aspects of health including elements of spiritual healing would likely be beneficial when working with this population.

## Data availability statement

The raw data supporting the conclusions of this article will be made available by the authors, without undue reservation.

## Ethics statement

The studies involving human participants were reviewed and approved by Protection of Human Rights in Research Committee, Biola University. The participants provided their written informed consent to participate in this study.

## Author contributions

KS has completed this project as part of her doctoral dissertation. DW, AC, and JP served as dissertation committee members, as well as principal investigators on the original grant for funding. RB and LH were instrumental in obtaining original grant funding. All authors contributed to the article and approved the submitted version.

## Funding

This study was funded by the Lily foundation grant initiative administered through the Association of Theological Schools.

## Conflict of interest

The authors declare that the research was conducted in the absence of any commercial or financial relationships that could be construed as a potential conflict of interest.

## Publisher’s note

All claims expressed in this article are solely those of the authors and do not necessarily represent those of their affiliated organizations, or those of the publisher, the editors and the reviewers. Any product that may be evaluated in this article, or claim that may be made by its manufacturer, is not guaranteed or endorsed by the publisher.
